# Antibiotic Resistance and Surgical Site Infections in Free Flap Reconstruction for Head and Neck Cancer: A Retrospective Analysis From a Lower-Middle-Income Country

**DOI:** 10.7759/cureus.82103

**Published:** 2025-04-11

**Authors:** Fizzah Arif, Safdar A Shaikh, Hibba E Arif, Haleema Sadia, Alizah Pervaiz Hashmi, Mohammad Fazlur Rahman

**Affiliations:** 1 Department of Plastic and Reconstructive Surgery, Aga Khan University Hospital, Karachi, PAK; 2 Department of Plastic Surgery, Aga Khan University Hospital, Karachi, PAK

**Keywords:** antimicrobial resistance, bacterial profile, free flap: oral cancer, ­reconstructive surgery, surgical site infection(ssi)

## Abstract

Objective: This study aimed to analyze the frequency of infection and its causative organisms, with their antibiotic susceptibility pattern, in patients who underwent free flap reconstructive surgery for head and neck cancers.

Methodology: This hospital record-based cross-sectional study was conducted at Aga Khan University Hospital in Karachi, Pakistan, involving 92 patients who underwent free flap reconstruction between January 1, 2023, and December 31, 2023. Data, including demographic information, surgical specifics, and microbial profiles, were collected from patient records, with a focus on postoperative wound infections after obtaining approval. Data analysis was done using IBM SPSS Statistics for Windows, Version 23 (Released 2015; IBM Corp., Armonk, New York, United States).

Results: Among 92 patients, 11 (11.9%) developed surgical site infections (SSIs). Male gender and age between 41 and 60 years were significantly associated with the occurrence of SSIs (p < 0.001). There was no significant relationship between age, American Society of Anesthesiologists (ASA) score, comorbidities, and flap types. Gram-negative bacteria, particularly *Pseudomonas aeruginosa*, dominated isolates. Early-onset SSIs were primarily associated with *P. aeruginosa*, whereas late-onset infections showed a broader spectrum of Gram-negative pathogens, including *Enterobacter *and *Klebsiella *species, suggesting temporal variations in microbial colonization. Multidrug-resistant organisms (MDROs) were identified in 66.6% of Gram-negative and all Gram-positive isolates, with extended drug resistance (XDRO) observed in select Gram-negative cases. *P. aeruginosa* was the most common Gram-negative isolate, while *Staphylococcus aureus* predominated among Gram-positive bacteria.

Conclusion: SSI was noted in 11.9% of the free flap population. Tailored antibiotic prophylaxis is crucial due to significant antimicrobial resistance in Gram-negative bacteria like *P. aeruginosa* and *Enterobacter*. Vigilant postoperative monitoring is essential given consistent pathogenic profiles in early- and late-onset infections, urging careful antibiotic stewardship in managing these infections effectively.

## Introduction

At 600,000 to 700,000 new cases reported globally each year, head and neck cancer ranks sixth globally in terms of cancer incidence [1,2]. The incidence of oral cancer has increased in both lower-middle-income (LMIC) and developed countries [3], and the overall incidence of head and neck cancer is expected to increase by 30% per year by 2030 [1,2]. Most of these cancers are squamous cell carcinomas (SCCs), which have a poor prognosis (around 50%) because of a higher chance of recurrence [4].

With a success rate of more than 95%, free-flap reconstruction is regarded as the gold standard of care for head and neck reconstruction, following complete resection [5]. Since most head and neck procedures damage the gastrointestinal tract while maintaining sterility, they are known to be clean-contaminated surgeries because they introduce oral flora and secretions from the oral cavity, increasing the risk of infection at the surgical site [6].

Surgical site infections (SSIs), reported in up to 10-45% of cases despite antibiotic prophylaxis, can lead to damage to the vascular supply of the area, resulting in free-flap failure. SSIs often precede and contribute to the development of oro-cutaneous fistulae (OCF), leading to an extended hospital stay, unexpected return to the operating room, need for additional surgery, and significant patient morbidity, including impaired oral intake, prolonged wound care, and reduced quality of life [5,6].

Most head and neck SSIs are caused by multi-microbial infections; however, others are caused by single Gram-negative bacteria such as *Pseudomonas aeruginosa* [7]. Effective use of antibiotic prophylaxis could dramatically lower SSI rates, as the incidence of SSI without prophylaxis has been reported to be as high as 87% [8].

In the past, the length of surgical prophylaxis was determined at the surgeon's discretion rather than being standardized or supported by evidence. Newer research, however, indicates that most infections may be avoided with a brief parenteral antibiotic course that lasts up to 24 hours postoperatively and begins with a single perioperative dosage [5].

Despite being one of the leading causes of free-flap failure, there is a lack of international and national guidelines for the dosage and duration of antibiotics to be administered in oral cancer cases requiring flap reconstruction. This cross-sectional study was carried out at a tertiary care facility in Karachi in order to ascertain the susceptibility profiles of the causative organisms in patients undergoing surgery for oral cancer, as well as the frequency of SSIs among patients undergoing free flap reconstruction following head and neck cancer surgery. It will aid in better understanding the type of organisms prevalent in our population, their susceptibility, and their resistance to antibiotics.

## Materials and methods

This hospital-based cross-sectional study received approval from the Ethics Review Committee, Aga Khan University Hospital and included data from all adult patients undergoing free flap reconstruction following biopsy-proven cancer excision in the head and neck region, between January 1, 2023, and December 31, 2023. Patients who developed wound infections and had samples from free flap wounds sent for culture and sensitivity within 10 days post-surgery were also eligible for inclusion. Patients were admitted to either the Otolaryngology or Plastic Surgery Department, Aga Khan University Hospital, one day before the surgery. Patients who experienced mortality during the postoperative period, underwent local flap reconstruction for head and neck conditions, or developed wound infection after 10 days were excluded from the study.

All patients received prophylactic antibiotics (cefazolin ± metronidazole) [7] preoperatively and continued postoperatively. Repeated intraoperative dosing was administered based on surgical duration exceeding four hours, per institutional protocol. Only oral sips of water were started on the third day. Percutaneous endoscopic gastrostomy/nasogastric (PEG/NG) tubes were placed intraoperatively for feeding. Antibiotic treatment spanned 3-5 days, with discharge typically on the fifth to sixth day. In the case of SSI, samples were sent for microbiological analysis; antibiotics were adjusted based on culture and sensitivity results and infectious disease consultation.

Data collection

Electronic patient medical records served as the data source, collected by medical professionals in January 2024, detailing patient demographics, SSIs, flap type, surgery duration, comorbidities, discharge day, postoperative infection onset, microbial profiles, and susceptibility profile to different antibiotics. Patient consent was obtained for the use of their data in this publication.

Data were analyzed using IBM SPSS Statistics for Windows, Version 23 (Released 2015; IBM Corp., Armonk, New York, United States). Descriptive statistics were employed to summarize the patient demographics, SSIs, microbial profiles, and antibiotic susceptibility patterns. Categorical variables were presented as frequencies and percentages, while continuous variables were expressed as means ± standard deviation (SD). The chi-square test was used to assess the association between categorical variables, such as the type of flap and the incidence of SSIs. A p-value less than 0.05 was considered statistically significant.

Pus swabs and aspirated samples from suspected SSIs were cultured using standard bacteriological methods, including Gram staining, biochemical testing, and colony morphology analysis.

## Results

Ninety-two patients in all received surgical procedures in this study, and they were all tracked during the investigation (Table 1).

**Table 1:** Characteristics of study participants Data are presented as frequencies for categorical variables. Statistical analysis was performed using the chi-square test. A p-value less than 0.05 was considered statistically significant. ASA: American Society of Anesthesiologists

Demographics	No. of flaps infected	Not infected	p-value
Age (years)			0.00
18-40	2	11	
41-60	9	51	
61-80	0	19	
>80	0	0	
Gender			0.00
Male	10	63	
Female	1	18	
ASA			0.49
I	0	5	
II	7	57	
≥III	4	19	
Prior co-morbidity			0.61
Yes	4	36	
No	7	45	
Type of free flap			0.72
Anterolateral thigh flap	4	33	
Fibula flap	5	24	
Radial forearm flap	2	23	
Lateral arm flap	0	1	

Out of these, 11 of 92 (11.9%) showed positive pus culture sent within the first 10 days of the procedure. Among infected patients, the mean age was 46.8 years ± 11.53 years. Two (18.2%) patients with flap infections were within the age range of 18-40 years, and 81.2% (n = 9) were aged 41-60 years. Notably, no infections occurred in patients older than 60 years. The age range of 41-60 years showed a higher percentage of SSIs. There was a statistically high significance between 41 and 60 years of age and flap infection, with p ≤ 0.001. There were 19 (20.7%) females and 73 (79.3%) males. Male gender was a highly significant risk factor for flap infection, with a p-value less than 0.001.

The rate of SSIs was 0% for patients with an ASA score of I, 10.9% (7 out of 64 patients) for those with an ASA score of II, and 17.4% (4 out of 23 patients) for those with an ASA score of III. The difference was not statistically significant (p = 0.49). Comorbidities were defined in this study as the co-occurrence of one or more additional disorders with the major concern disease at the time of surgery. Consequently, four (36.3%) patients with flap infections had co-morbid conditions overall, while seven (63.6%) patients without comorbidities had SSIs, which was not statistically significant (p = 0.61).

Among the flap types, the anterolateral thigh flap was the most common (37, 40.2%), followed by the free fibula flap (29, 31.5%), the radial forearm flap (25, 27.1%), and the lateral arm flap (1, 1.1%). The rate of SSIs in the free fibula flap was highest (5, 45.5%), followed by four (36.4%) in the anterolateral thigh flap and two (18.2%) in the radial forearm flap. No infection developed in the lateral arm flap. There was no statistically significant relationship between types of flaps and the development of SSI (p = 0.72).

Bacterial isolates

Wound swabs were obtained strictly from the 11 patients in the study group who developed SSIs post-head and neck reconstruction with free flaps, and the samples were cultured using standard bacteriological methods. Eight distinct bacterial isolates and two distinct fungal isolates were obtained from all SSI patients who had at least one bacterial growth that tested positive for culture.

Table 2 illustrates the distribution of Gram-positive and Gram-negative bacteria in different plastic surgery procedures; the difference between these was not statistically significant (p > 0.05).

**Table 2:** Pathogens isolated according to flap type *S. aureus*: *Staphylococcus aureus*; *Strep​*​​​​​​. *milleri*: *Streptococci* *milleri*; *E. coli*: *Escherichia coli*; BHS:beta hemolytic *Streptococci*; Candida A: *Candida albicans*; Candida G: *Candida glabrata*

	Gram positive				Gram negative				Fungus	
Types of free flaps	S. aureus	Enterococci	Strep. milleri	BHS Group G	Klebsiella	Pseudomonas	E. coli	Enterobacter	*Candida *G	*Candida *A
Anterolateral thigh flap	1	0	0	1	0	2	1	1	0	1
Fibula flap	0	1	1	0	1	3	0	2	0	1
Radial forearm flap	1	1	0	0	0	2	0	0	1	0
Total	2	2	1	1	1	7	1	3	1	2

Out of a total of 21 isolates, 57.1% (12/21) were Gram-negative, 28.6% (6/21) were Gram-positive, and 14.3% (3/21) were fungal isolates. Among the types of bacteria identified, *P. aeruginosa* was the most common at 33.3% (7/21), followed by *Enterobacter *at 14.2% (3/21), *Staphylococcus aureus*, *Enterococcus*, and *Candida albicans* each at 9.5% (2/21), *Streptococci milleri*, beta hemolytic *Streptococci *group G (BHS Group G), *Klebsiella *species, *Escherichia coli*, and *Candida glabrata* each at 4.8% (1/21). Table 2 displays the distribution of pathogenic bacteria throughout different plastic surgery operations.

Onset of infection

In this study, 11 cases of SSIs were identified, with infections categorized based on their onset. Early infections, defined as those occurring within the first four days post-surgery, were observed in five cases (45.5%). These included two cases in anterolateral thigh flaps, two in free fibula flaps, and one in a radial forearm flap. Late infections, occurring after the fourth postoperative day, were recorded in six cases (54.5%), comprising three cases in free fibula flaps, two in anterolateral thigh flaps, and one in a radial forearm flap. The distribution of early and late infections across flap types did not differ significantly (p = 0.99), as shown in Table 3.

**Table 3:** Onset of infection based on flap type Data were presented as frequencies for categorical variables. Statistical analysis was performed using the chi-square test for categorical variables, with statistical significance defined as a p-value < 0.05.

Types	Early onset of infection within or on 4th day		Total	p-value
	Yes	No		0.99
Anterolateral thigh flap	2	2	4	
Fibula flap	2	3	5	
Radial forearm flap	1	1	2	
Total	5	6	11	

In this study, among 21 pathogens causing infection in 11 cases, nine (42.8%) pathogens caused infection within four days after surgery (early infection), and 12 (57.1%) pathogens caused infection after four days of surgery (late infection). Table 4 displays the pathogenic bacterial distribution in early and late infection, and the difference was not statistically significant (p > 0.05).

**Table 4:** Types of isolated micro-organisms according to onset of infection

S. No.	Microorganism	Early onset of infection within or on the 4th day		p-value
		Yes (n)	No (n)	
	Gram-positive pathogens			0.87
1	Staphylococcus aureus	1	1	
2	Enterococci	1	1	
3	Streptococci milleri	-	1	
4	* *Beta hemolytic *Streptococci *Group G	-	1	
	Gram-negative pathogens			0.45
1	Pseudomonas aeruginosa	4	3	
2	* Enterobacter *species	-	3	
3	Klebsiella pneumoniae	-	1	
4	Escherichia coli	1	-	
	Fungal			0.37
1	Candida glabrata	1	-	
2	Candida albicans	1	1	

Antimicrobial sensitivity and resistance profiles

Antimicrobial sensitivity testing revealed 100% susceptibility of *S. aureus* to vancomycin, clindamycin, and trimethoprim/sulfamethoxazole, followed by 50% susceptibility of *S. aureus* to erythromycin, levofloxacin, and gentamicin.

*Enterococcus *species (2, 100%) were fully sensitive to vancomycin and chloramphenicol, and one (50%) was sensitive to linezolid, erythromycin, and ampicillin. One (100%) *S. milleri* and one (100%) BHS Group B showed sensitivity toward vancomycin, ofloxacin, clindamycin, penicillin G, and ceftriaxone.

Gram-negative bacteria, particularly *P. aeruginosa*, demonstrated (7, 100%) sensitivity to amikacin, ciprofloxacin, imipenem, meropenem, piperacillin-tazobactam, gentamicin, and ceftazidime, while *Klebsiella pneumoniae* (1, 100%) and *Enterobacter *(3, 100%) were sensitive to amikacin, ciprofloxacin, imipenem, meropenem, piptaz, aztreonam, gentamicin, ceftriaxone, cefuroxime, cefixime, and cotrimoxazole. These findings are summarized in Tables 5-6.

**Table 5:** Antibiotic susceptibility patterns of Gram-positive bacteria isolated from wound samples

Sensitivities of antibiotics for Gram-positive bacteria				
Antibiotics tested	*Staphylococcus aureus *(n = 2) % Susceptible	*Enterococcus *(n = 2) % Susceptible	Beta-hemolytic *Streptococcus* group (n = 1) % Susceptible	*Streptococcus milleri* (n = 1) % Susceptible
Vancomycin	2 (100%)	2 (100%)	1 (100%)	1 (100%)
Linezolid	-	1 (50%)	-	-
Erythromycin	1 (50%)	1 (50%)	-	-
Levofloxacin	1 (50%)	-	-	-
Ofloxacin	-	-	1 (100%)	1 (100%)
Penicillin G	-	-	1 (100%)	1 (100%)
Gentamicin	1 (50%)	-	-	-
Clindamycin	2 (100%)	-	1 (100%)	1 (100%)
Chloramphenicol	-	2 (100%)	-	-
Ceftriaxone	-	-	1 (100%)	1 (100%)
Ampicillin	-	1 (50%)	-	-
Trimethoprim/sulfame	2 (100%)	-	-	-

**Table 6:** Antibiotic susceptibility patterns of Gram-negative bacteria isolated from wound sample

Sensitivities of antibiotics for Gram-negative bacteria				
Antibiotics tested	*Pseudomonas aeruginosa* (n = 7) % Susceptible	*Enterobacter *(n = 3) % Susceptible	*Klebsiella *(n = 1) % Susceptible	*Escherichia coli* (n = 1) % Susceptible
Amikacin	7 (100%)	3 (100%)	1 (100%)	1 (100%)
Amoxicillin/Clavulanic acid	-	-	-	1 (100%)
Piptaz	7 (100%)	3 (100%)	1 (100%)	1 (100%)
Imipenem	7 (100%)	3 (100%)	1 (100%)	1 (100%)
Meropenem	7 (100%)	3 (100%)	1 (100%)	1 (100%)
Ciprofloxacin	7 (100%)	3 (100%)	1 (100%)	-
Aztreonam	5 (71.4%)	3 (100%)	1 (100%)	1 (100%)
Gentamicin	7 (100%)	3 (100%)	1 (100%)	-
Ceftazidime	7 (100%)	-	-	-
Tobramycin	3 (42.9%)	-	-	-
Ceftriaxone	-	3 (100%)	1 (100%)	-
Cefuroxime	-	3 (100%)	1 (100%)	-
Cefixime	-	3 (100%)	1 (100%)	-
Co-trimoxazole	-	3 (100%)	1 (100%)	-

This study examined antibiotic resistance patterns in bacteria isolated from SSIs (Table 7).

**Table 7:** Antibiotic resistance pattern of isolated bacteria from wound ND: not done

	Resistant Gram-positive bacteria		Resistant Gram-negative bacteria			
Antibiotics tested	*Staphylococcus aureus* (n = 2) % Resistant	*Enterococcus *(n = 2) % Resistant	*Pseudomonas aeruginosa* (n = 7) % Resistant	*Enterobacter *(n = 3) % Resistant	*Klebsiella *(n = 1) % Resistant	Escherichia coli (n = 1) % Resistant
Amoxicillin/Clavulanic acid	-	-	ND	3 (100%)	1 (100%)	-
Cotrimoxazole	-	-	ND	-	-	1 (100%)
Erythromycin	1 (50%)	1 (50%)	ND	-	-	-
Levofloxacin	1 (50%)	-	ND	-	-	-
Ciprofloxacin	-	-	ND	-	-	1 (100%)
Penicillin G	1 (50%)	-	ND	-	-	-
Gentamicin	1 (50%)	-	ND	-	-	1 (100%)
Tetracycline	2 (100%)	2 (100%)	ND	-	1 (100%)	-
Oxacillin	2 (100%)	-	ND	-	-	-
Fusidic acid	2 (100%)	-	ND	-	-	-
Ampicillin	-	1 (50%)	ND	3 (100%)	1 (100%)	1 (100%)
Ceftriaxone	-	-	ND	-	-	1 (100%)
Cefuroxime	-	-	ND	-	-	1 (100%)

All Gram-negative isolates (n = 12) demonstrated 100% resistance to ampicillin. Additionally, *Enterobacter *exhibited 100% resistance to amoxicillin-clavulanic acid along with ampicillin, while *Klebsiella *showed resistance to tetracycline and amoxicillin-clavulanic acid, alongside ampicillin. *E. coli* (1, 100%) isolates were resistant to cotrimoxazole, ciprofloxacin, gentamicin, tetracycline, ceftriaxone, and cefuroxime. Multidrug resistance (MDR), defined as resistance to three or more antibiotic classes, was observed in 66.6% (2/3) of Gram-negative isolates.

Among Gram-positive isolates, *S. aureus* displayed the highest resistance to tetracycline (2, 100%), followed by erythromycin, oxacillin, and fusidic acid (1, 50%). Additionally, *S. aureus* (1, 50%) exhibited partial resistance to gentamicin, levofloxacin, and penicillin G. One out of two (1, 50%) isolates of *Enterococcus *showed resistance to erythromycin, tetracycline, and ampicillin. None of the isolates exhibited resistance to piptaz, imipenem, meropenem, or amikacin. MDR was noted in 100% (2/2) of Gram-positive species.

## Discussion

In 92 cases of free flap surgeries performed for head and neck cancers, an 11.9% incidence of SSI was noted. In an Indian prospective study of wound infection in oral cancer, 61% of patients had positive culture [9]. The incidence of SSIs was 10.9% in research from Rwanda, 15.9% in major oral oncology surgery in China, 11% in the National Surgical Quality Improvement Program (NSQIP) database of Lebanon from 2005 to 2020, and 20.4% in Australia [10-13].

A higher percentage of infection was identified among patients aged 41-60 years, with statistically significant implications. Additionally, the risk of SSI was found to increase with age, particularly for individuals older than 45 years [12]. In our study, the apparent higher risk in the 41-60 year age group may partly reflect the larger proportion of cases in that demographic, rather than an age-specific predisposition. Conversely, a study suggested the highest probability of SSI was in the 16-24 age category [14]. ASA class III-IV was found to be a major risk factor for SSI after head and neck surgery [11], although other studies reported no significant impact of advanced ASA on postoperative wound infection development [13,15]. In line with prior research, this study did not establish a statistically significant correlation between SSIs and comorbidities, highlighting the need for further investigation into potential causes [11,16,17].

Among the types of flaps, free fibula flaps exhibited the highest incidence of SSIs - 17.2% in our study, consistent with another study noting osseous vascularized flaps as a risk for postoperative SSIs [18]. This group commonly involved the use of implants for bone fixation, which may have contributed to the higher infection rates. However, specific data on implant use in the study population was not collected, which limits further analysis. Conversely, an alternate study identified the anterolateral thigh flap as a separate risk factor for SSIs in contrast to other free flaps [16].

The research revealed a varied range of pathogens among SSI bacterial isolates, with Gram-negative bacteria being the most prevalent. This contradicts previous findings that identified Gram-positive bacteria as common postoperative infective agents [13,15,19], given that the upper aerodigestive tract (UADT) predominantly contains Gram-positive flora, along with fewer commensals of facultative anaerobes and, to a lesser extent, fungal and Gram-negative bacteria [20].

Research on the prophylactic antibiotics in head and neck cancer procedures, particularly in free flap reconstructions, revealed a greater prevalence of SSIs when Clindamycin was used as an antibiotic [15,20]. Regarding the organisms isolated from our study population, the most prevalent Gram-positive bacteria were *S. aureus*, and the most common Gram-negative bacteria were *P. aeruginosa*. Similarly, another study reported S. aureus [19,21] and *P. aeruginosa* as the most cultured pathogens [21]. *Streptococcus *spp. was seen to be highest in a few studies [13,15]. Studies reporting *S. aureus* used clindamycin and cefazolin or Augmentin for prophylaxis [19,21], whereas *Streptococcus *was highest in patients who were administered cefazolin and metronidazole for prophylaxis in two studies [13,15]. *Pseudomonas *infection was highest with Clindamycin-Augmentin prophylaxis [21]; however, in our patients with cefazolin ± metronidazole prophylaxis, *Pseudomonas *was still the highest.

Methicillin-resistant *S. aureus *(MRSA) is reported in head and neck cancer patients at a prevalence ranging from 6% to 20% in various studies [22]. In our study, *S. aureus* was isolated in two cases, both of which were identified as MRSA. This 100% MRSA rate among *S. aureus* isolates highlights the potential challenges of managing SSIs in our patient population.

Another study reported similar findings to our study, indicating that mostly Gram-negative organisms were isolated from SSIs of UADT reconstructive surgeries, which are not typical oral commensals but can colonize UADT due to poor general health [18,20]. Different prophylactic antibiotics were administered preoperatively in this study; however, preoperative topical antisepsis, mucosal preparation, and systemic prophylaxis with piperacillin and tazobactam were significantly linked to a lower incidence of infection in this trial [18]. It is important to note that while metronidazole is effective against anaerobic bacteria and certain protozoa, it lacks efficacy against aerobic Gram-negative bacteria like *Pseudomonas *[5,20].

A study that compared patient postoperative infection rates, undergoing free flap reconstruction with or without anaerobic coverage, did not find any statistical differences. Remarkably, patients with anti-*Pseudomonas *coverage had a higher risk of postoperative infections, whereas those without it had a higher risk of *Pseudomonas *growth. However, our overall *Pseudomonas *infection rate was notably high at 63.6% (7 out of 11 positive cultures), in contrast to other studies reporting rates between 9%, 11.3%, and 13% [22-24]. *Pseudomonas *is known for its substantial mortality impact on cancer patients. The elevated incidence underscores the importance of incorporating antibiotics such as ciprofloxacin, levofloxacin, ceftazidime, cefepime, or carbapenems in the prophylaxis regimen [22].

In our research, we found that bacterial isolates causing SSIs had high rates of resistance to widely used medicines. Another study analyzing antimicrobial resistance in patients with free flap wound infections reported a different pattern of drug resistance among Gram-negative species compared to our population. Similarly, Gram-positive isolates of *S. aureus *displayed some resistance to clindamycin, oxacillin, erythromycin, cotrimoxazole, and levofloxacin, while *Enterococcus *showed resistance to Tetracycline [22]. These findings were somewhat like our results, with no evidence of MDR involving three or more classes of antibiotics noted.

Amoxicillin-clavulanate, ampicillin-sulbactam, and cefazolin are the recommended antibiotics for clean-contaminated head and neck surgery, according to a systematic review of 39 trials. In cases of penicillin allergy, ampicillin-sulbactam is recommended over clindamycin due to an increased risk of wound infection [20]. Because of their comprehensive coverage against aerobic Gram-positive and certain Gram-negative bacteria, first-generation cephalosporins are chosen to avoid SSIs in general surgery. Future research should focus on finding optimal alternatives for penicillin-allergic patients [18,20].

Numerous studies highlight how crucial anaerobic coverage is while using flap repair in head and neck surgery, as anaerobic bacteria constitute a significant portion of the flora in this region [25-28]. Current antibiotic regimens should ideally be administered within 60 minutes before incision, repeated during surgeries lasting over 4 hours, and continued for 24 hours. Some evidence suggests extending administration to 48 hours, but doing so does not further reduce infection rates [20,29] and is connected to higher chances of negative consequences, like complications brought on by antibiotics [20].

The findings of this study would help in influencing healthcare policies, leading to changes in training requirements or institutional guidelines, thereby improving the overall standard of care. Similar to other international studies [21-24], the identification of *Pseudomonas *as the most common organism in SSIs in our study highlights the need for targeted infection prevention strategies, such as tailored antimicrobial prophylaxis and enhanced hospital and hand hygiene, infection control protocols, and early detection of hospital-acquired infections. This knowledge can also guide patient education and postoperative care to reduce infection risks. Ultimately, modifying practices based on this finding can help decrease *Pseudomonas*-related SSIs and improve patient outcomes.

Limitations

The limited sample size of 92 free flaps for head and neck cancers may restrict the generalizability of findings to a broader patient population. The study design's hospital record-based cross-sectional nature introduces inherent limitations, as it relies on existing data and may be susceptible to biases and incomplete information. The absence of a standardized protocol for antibiotic prophylaxis across all cases could introduce variability in the study results. The absence of data on comorbidities such as smoking, diabetes, and preoperative radiation, which are potential risk factors for developing infections, is an additional limitation. Therefore, we suggest that future prospective studies with larger, more heterogeneous cohorts and standardized protocols are warranted to validate these results.

## Conclusions

With approximately one in every 10 free flaps developing an SSI, this study underscores the critical need for tailored antibiotic prophylaxis guided by the predominant pathogens, particularly in light of the high rates of antimicrobial resistance observed among Gram-negative bacteria such as *P. aeruginosa *and *Enterobacter*. No significant association was found between types of flaps and SSI rates. The distribution of early and late-onset infections did not differ significantly in terms of pathogenic bacterial profiles, highlighting the importance of consistent postoperative monitoring. The varying antimicrobial resistance patterns among bacterial isolates stress the necessity for judicious antibiotic use to effectively manage SSIs following free flap reconstruction for head and neck cancer.

## References

[1] Sung H, Ferlay J, Siegel RL, Laversanne M, Soerjomataram I, Jemal A, Bray F (2021). Global cancer statistics 2020: GLOBOCAN estimates of incidence and mortality worldwide for 36 cancers in 185 countries. CA Cancer J Clin.

[2] Fitzmaurice C, Allen C, Barber RM (2017). Global, regional, and national cancer incidence, mortality, years of life lost, years lived with disability, and disability-adjusted life-years for 32 cancer groups, 1990 to 2015: a systematic analysis for the global burden of disease study. JAMA Oncol.

[3] Bravi F, Lee YA, Hashibe M (2021). Lessons learned from the INHANCE consortium: an overview of recent results on head and neck cancer. Oral Dis.

[4] Pang L, Jeannon JP, Simo R (2011). Minimizing complications in salvage head and neck oncological surgery following radiotherapy and chemo-radiotherapy. Curr Opin Otolaryngol Head Neck Surg.

[5] Haidar YM, Tripathi PB, Tjoa T (2018). Antibiotic prophylaxis in clean-contaminated head and neck cases with microvascular free flap reconstruction: a systematic review and meta-analysis. Head Neck.

[6] Kinzinger MR, Bewley AF (2017). Perioperative care of head and neck free flap patients. Curr Opin Otolaryngol Head Neck Surg.

[7] Bratzler DW, Dellinger EP, Olsen KM (2013). Clinical practice guidelines for antimicrobial prophylaxis in surgery. Surg Infect (Larchmt).

[8] Khariwala SS, Le B, Pierce BH, Vogel RI, Chipman JG (2016). Antibiotic use after free tissue reconstruction of head and neck defects: short course vs long course. Surg Infect (Larchmt).

[9] Balagopal PG, Suresh S, Nath SR, Sagila SG, George NA (2022). Pattern of post-operative infections among oral cavity cancer patients in a tertiary care cancer Centre: a prospective study. Indian J Otolaryngol Head Neck Surg.

[10] Mukagendaneza MJ, Munyaneza E, Muhawenayo E (2019). Incidence, root causes, and outcomes of surgical site infections in a tertiary care hospital in Rwanda: a prospective observational cohort study. Patient Saf Surg.

[11] Hassan B, Abou Koura A, Makarem A, Abi Mosleh K, Dimassi H, Tamim H, Ibrahim A (2023). Predictors of surgical site infection following reconstructive flap surgery: a multi-institutional analysis of 37,177 patients. Front Surg.

[12] Zhang L, Zheng J, Mu J, Gao Y, Li G (2022). Risk factors for postoperative complications following aesthetic breast surgery: a retrospective cohort study of 4973 patients in China. Aesthetic Plast Surg.

[13] Daly JF, Gearing PF, Tang NS, Ramakrishnan A, Singh KP (2022). Antibiotic prophylaxis prescribing practice in head and neck tumor resection and free flap reconstruction. Open Forum Infect Dis.

[14] Mohan N, Gnanasekar D, Tk S, Ignatious A (2023). Prevalence and risk factors of surgical site infections in a teaching Medical College in the Trichy district of India. Cureus.

[15] Gearing PF, Daly JF, Tang NS, Singh K, Ramakrishnan A (2021). Risk factors for surgical site infection in free-flap reconstructive surgery for head and neck cancer: retrospective Australian cohort study. Head Neck.

[16] Shi M, Han Z, Qin L (2020). Risk factors for surgical site infection after major oral oncological surgery: the experience of a tertiary referral hospital in China. Int Med Res.

[17] Iliopoulou-Kosmadaki S, Hadjimichael AC, Kaspiris A (2023). Impact of preoperative quality of life and related factors on the development of surgical site infections following primary total joint arthroplasty: a prospective case-control study with a five-year follow-up. Adv Orthop.

[18] Beydoun AS, Koss K, Nielsen T (2022). Perioperative topical antisepsis and surgical site infection in patients undergoing upper aerodigestive tract reconstruction. JAMA Otolaryngol Head Neck Surg.

[19] Orabona GD, Corvino R, Maglitto F (2018). Infective complications after free flaps reconstruction in patients affected by head and neck cancer: our experience on 77 cases. Annali Italiani Di Chirurgia.

[20] Vander Poorten V, Uyttebroek S, Robbins KT (2020). Perioperative antibiotics in clean-contaminated head and neck surgery: a systematic review and meta-analysis. Adv Ther.

[21] Yang S, Nadimi S, Eggerstedt M, Thorpe E, Pittman AJHNCR (2016). Antibiotic prophylaxis and postoperative wound infection rates in salvage surgery for head and neck cancer. Head Neck Cancer Res.

[22] Hamill CS, Snyder V, Sykes KJ, O'Toole T (2021). Prevalence of multidrug-resistant organisms in patients undergoing free flap reconstruction. Laryngoscope.

[23] Wagner JL, Kenney RM, Vazquez JA, Ghanem TA, Davis SL (2016). Surgical prophylaxis with gram-negative activity for reduction of surgical site infections after microvascular reconstruction for head and neck cancer. Head Neck.

[24] Bartella AK, Kamal M, Teichmann J, Kloss-Brandstätter A, Steiner T, Hölzle F, Lethaus B (2017). Prospective comparison of perioperative antibiotic management protocols in oncological head and neck surgery. J Craniomaxillofac Surg.

[25] Robbins KT, Byers RM, Cole R (1988). Wound prophylaxis with metronidazole in head and neck surgical oncology. Laryngoscope.

[26] Veve MP, Davis SL, Williams AM, McKinnon JE, Ghanem TA (2017). Considerations for antibiotic prophylaxis in head and neck cancer surgery. Oral Oncol.

[27] Veve MP, Greene JB, Williams AM (2018). Multicenter assessment of antibiotic prophylaxis spectrum on surgical infections in head and neck cancer microvascular reconstruction. Otolaryngol Head Neck Surg.

[28] Callender DL (1999). Antibiotic prophylaxis in head and neck oncologic surgery: the role of Gram-negative coverage. Int J Antimicrob Agents.

[29] Balamohan SM, Sawhney R, Lang DM (2019). Prophylactic antibiotics in head and neck free flap surgery: a novel protocol put to the test. Am J Otolaryngol.

